# 

**Published:** 1899-03

**Authors:** 


### THE BRISTOL
### Medico-Chirurgical Journal.

A JOURNAL OF THE MEDICAL SCIENCES FOR THE WEST OF ENGLAND AND SOUTH WALES

*PUBLISHED UNDER THE AUS ICES OF*

*THE BRISTOL MEDICO-CHIRURGICAL SOCIETY.*

21

EDITOR:

R. SHINGLETON SMITH, M.D., B.Sc.,

WITH WHOM ARE ASSOCIATED

J. MICHELL CLARKE, M.A., M.D., W. H. HARSANT,
B. M. H. ROGERS, B.A., M.D., B.CH., AND JAMES SWAIN, M.S., M.D.

ASSISTANT-EDITOR:

L. M. GRIFFITHS.

EDITORIAL SECRETARY:

JAMES TAYLOR.

---

"Scire est nescire, nisi id me

Scire alius sciret."

---

JUN -3 1901
174061.

*VOL. XVII.*

BRISTOL: J. W. ARROWSMITH.
LONDON: J. & A. CHURCHILL, 7 GREAT MARLBOROUGH STREET.
1899.

WI
BR323

J. W. Arrowsmith

Publisher Bristol

### CONTENTS OF VOLUME XVII.

*Page.*

The Surgical Treatment of Myoma of the Uterus. By W. ROGER WILLIAMS, F.R.C.S. ... ... ... ... ... ... ... ... ... ... 1

A Case of Hysterectomy for Fibroid Tumour of Uterus, with Intra-Abdominal Treatment of Pedicle. By W. H. C. NEWNHAM, M.A., M.B. Cantab. ... ... ... ... ... ... ... ... ... ... 8

Symphysiotomy. By WALTER C. SWAYNE, M.D. Lond... ... ... 11

A Case of Acute Ulcerative Endocarditis. By H. L. ORMEROD, M.D.R.U.I., M.R.C.S., L.R.C.P. ... ... ... ... ... ... ... 15

Achondroplasia. By CHARLES E. S. FLEMMING, M.R.C.S., L.R.C.P. 21

The Bacteriology of Some Suppurations Complicating Pulmonary DIsease. By J. O. SYMES, M.D., D.P.H. ... ... .. ... ... ... 30

On the Temperature in Cases of Apoplexy, and on the Occurrence (1) of Œdema and (2) of Loss of the Knee-Jerk in the Paralysed Limbs in Hemiplegia. By J. MICHELL CLARKE, M.A., M.D. Cantab., F.R.C.P. ... ... ... ... ... ... ... ... ... ... ... 97

Fracture of the Patella. By A. W. PRICHARD, M.R.C.S. ... ... 104

Idiopathic Thrombosis of the Portal and Contributory Veins. By BERTRAM M. H. ROGERS, B.A., M.D., B.Ch. Oxon. ... ... ... 107

A Case of Extreme Bradycardia. By R. GEORGE FENDICK, M.R.C.S., and GEORGE PARKER, M.D. Cantab. With Notes on the Eye-Symptoms by F. RICHARDSON CROSS, M.B. Lond., F.R.C.S. ... 113

A Case of Dislocation of the Hand and Carpus Backwards. By TRAFFORD MITCHELL, M.D. Glasg. ... ... ... ... ... ... ... 121

The Antiseptic and Disinfectant Properties of Soap. By J. O. SYMES, M.D. Lond., D.P.H. ... ... ... ... ... ... ... ... ... ... 193

*Page.*

On the Treatment of Subacute Bronchitis. By F. H. Edgeworth, M.B., B.Sc., B.A. Cantab. ... ... ... ... ... ... ... ... ... 197

Some Groups of Interesting Cases of Abdominal Surgery. By Charles A. Morton, F.R.C.S. Eng. ... ... ... ... ... ... 203

Some Unusual Operations on Lunatics and their Results. By J. Paul Bush, M.R.C.S. Eng. ... ... ... ... ... ... ... ... 219

A Simple Form of Influence Machine for X-Ray Work. By William Cotton, M.A., M.D. Ed. ... ... ... ... ... ... ... ... ... 222

Medical Bristol in the Eighteenth Century. By W. H. Harsant, F.R.C.S. Eng. ... ... ... .. ... ... ... ... ... ... ... ... 297

The Varieties of Uterine Neoplasms and their Relative Frequency. By W. Roger Williams, F.R.C.S. Eng. ... ... ... ... ... 314

A Case of Neuritic Muscular Atrophy ("Peroneal" Type). By J. E. Shaw, M.B. Ed. ... ... ... ... .. ... ... ... ... ... 319

Irreducible Intussusception: with Notes on a Fatal Case. By G. L. Kerr Pringle, M.D. Ed. ... ... ... ... ... ... ... ... ... 322

Progress of the Medical Sciences ... ... ... 36, 121, 236, 326

Reviews of Books ... ... ... ... ... .. ... ... 50, 138, 254, 342

Notes on Preparations for the Sick ... ... ... 77, 160, 280, 366

Meetings of Societies ... ... ... ... ... ... ... 79, 161, 367

The Library of the Bristol Medico-Chirurgical Society ... ... ... ... ... ... ... ... ... 89, 177, 282, 370

Local Medical Notes ... ... ... ... ... ... ... 92, 181, 287, 374

Scraps ... ... ... ... ... ... ... ... ... ... ... 93, 190, 295, 381

### The Library of the Bristol Medico-Chirurgical Society.

*The following donations have been received since the publication of the list in December:*

*February 28th, 1899.*

| | |
|---|---|
| Clinical Society of London (1) ... ... ... ... | 1 volume. |
| Laryngological Society of London (2) ... ... ... ... | 1 ,, |
| B. M. H. Rogers, M.D. ... ... ... ... ... ... ... | 3 volumes. |
| Royal Academy of Medicine in Ireland ... ... | 1 volume. |
| Surgeon-General, United States Army (3) ... ... | 14 volumes. |
| James Swain, M.D. ... ... ... ... ... ... ... ... ... | 1 volume. |
| Caleb Trapnell (4) ... ... ... ... ... ... ... ... | 1 ,, |

Unbound periodicals have been received from Dr. B. M. H. Rogers, Dr. R. Shingleton Smith, the Surgeon-General, United States Army, and Dr. B. W. Walker. Dr. Shaw has presented a framed picture; and unframed pictures have been received from the Surgeon-General, United States Army, and Dr. De Forest Willard.

### THIRTY-FIRST LIST OF BOOKS.

The titles of books mentioned in previous lists are not repeated.

The figures in brackets refer to the figures after the names of the donors, and show by whom the volumes were presented. The books to which no such figures are attached have either been bought from the Library Fund or received through the *Journal*.

| | | |
|---|---|---|
| **Aarons, S. J.** ... | *Golden Rules of Gynæcology* ... ... ... ... ... ... ... | [1898] |
| **Bannatyne, G. A.** | *The Thermal Waters of Bath* ... ... ... ... ... ... | 1899 |
| **Beasley, H.** ... ... | *The Pharmaceutical Formulary.* 12th Ed. (Ed. by J. O. Braithwaite) ... ... ... ... ... ... | 1899 |

**Bell, J.** ... ... ... *Notes on Surgery for Nurses.* ... ... ... ... ... 5th Ed. 1899

**Burdon-Sanderson, J.** *Ludwig and Modern Physiology* ... ... ... ... ... 1898

*Catalogue (Index) of the Library of the Surgeon-General's Office, United States Army.* 2nd Ser., Vol. III. ... ... (3) 1898

*Children, An American Text-Book of the Diseases of.* (Ed. by L. Starr and T. S. Westcott.) 2nd Ed. 2 parts ... ... 1898

**Copeman, S. M.** ... *Vaccination: its Natural History and Pathology* ... ... 1899

**Dowse, T. S.** ... *The Treatment of Disease by Physical Methods* ... ... 1898

**Engelmann, G. J.** *Accouchements chez les Peuples primitifs.* (Étude français par P. Rodet) ... ... ... ... (4) 1886

**Fenwick, E. H.** ... *Golden Rules of Surgical Practice.* ... ... ... 5th Ed. [1898]

**Foster, M.** ... ... *Recent Advances in Science* ... ... ... ... ... ... ... 1898

**Fothergill, W. E.** *Golden Rules of Obstetric Practice* ... ... ... ... ... [1898]

**Gage, S. H.** ... ... *The Processes of Life revealed by the Microscope* ... ... 1898

*Glycerine, The Properties and Uses of Pure* ... ... ... ... ... ... ... ... [1898]

**Gowers, Sir W. R.** *Diseases of the Nervous System.* 3rd Ed. (Ed. by Sir W. R. Gowers and J. Taylor.) Vol. I. ... 1899

**Grünwald, L.** ... *Atlas and Abstract of the Diseases of the Larynx.* (Translated. Ed. by C. P. Grayson) ... ... 1898

**Guy de Chauliac...** *La Grande Chirurgie, 1363.* (Revue par E. Nicaise) 1890

*Gynecology, An American Text-Book of.* (Ed. by J. M. Baldy.) 2nd Ed. 1898

**Haviland, A.** ... *The Geographical Distribution of Disease in Great Britain.* ... ... ... ... ... ... ... ... 2nd Ed. 1892

**Haydn, J.** ... ... *The Book of Dignities.* 3rd Ed. (Ed. by H. Ockerby.) 1894

**Hewlett, R. T.** ... *A Manual of Bacteriology* ... ... ... ... ... ... ... 1898

**Hillemand, C.** ... *Organothérapie ou Opothérapie* ... ... ... ... ... ... 1899

**Hofmann, E. von** *Atlas of Legal Medicine.* (Translated. Ed. by F. Peterson and A. O. J. Kelly) ... ... ... ... 1898

**Keen, W. W.** ... *The Surgical Complications and Sequels of Typhoid Fever* 1898

**Kingscote, E.** ... *On So-called Spasmodic Asthma* ... ... ... ... ... ... 1899

*Lexicon of Medicine and the Allied Sciences.* (New Syd. Soc.) Part XXIV. 1898

**Liaras, E. J. Moure et G.** *Traitement chirurgical de quelques Paralysies faciales d'Origine otique* ... ... ... ... ... ... 1899

**Longmore, Sir T.** *Richard Wiseman* ... ... ... ... ... ... ... ... ... 1891

**Macilwain, G.** ... *Memoirs of John Abernethy.* 2 vols. ... ... ... ... 1853

**M'Keown, W. A....** *"Unripe" Cataract* ... ... ... ... ... ... ... ... 1898

**Méric, H. de** ... *Dictionary of Medical Terms—English-French* ... ... 1899

**Mondeville, H. de** *Chirurgie, 1306 à 1320.* (Traduction française, avec des notes, par E. Nicaise) ... ... ... 1893

**Moure et G. Liaras, E. J.** *Traitement chirurgical de quelques Paralysies faciales d'Origine otique* ... ... ... ... ... ... 1899

**Mraček, F.** ... ... *Atlas of Syphilis and the Venereal Diseases.* (Translated. Ed. by L. B. Bangs) ... ... ... ... 1898

*Myxœdema, Report of a Committee of the Clinical Society of London on...* (1) 1888

**Nahm, [—]** ... ... *The Erection of a Consumptive Sanatorium for the People.* (Tr. by W. Calwell) ... ... ... ... 1898

**Nisbet, W. W. Van Valzah and J. D.** [Eds.] *The Diseases of the Stomach* 1899

*Pathology, An Atlas of Illustrations of.* (New. Syd. Soc.) Fasc. XII. ... 1898

**Powell, Sir R. D....** *Treatment in Diseases and Disorders of the Heart* ... 1899

**Prichard, J. C.** ... *Physical History of Mankind.* Vols. I., 4th Ed., 1841; II.–IV., 3rd Ed., 1837–44; V. 1847

**Robinson, H. B.** ... *Traumatic Birth Paralyses of the Upper Extremity* ... 1899

**Roth, B.** ... ... *Lateral Curvature of the Spine.* ... ... ... 2nd Ed. 1899

**Schweinitz, E. A. de** *The War with the Microbes* ... ... ... ... ... 1898

**Thorne, W. B.** ... *Observations on Cardio-Vascular Repair* ... ... ... 1898

**Thurston, R. H.** ... *The Animal as a Prime Mover* ... ... ... ... ... 1898

**Tirard, N.** ... ... *Albuminuria and Bright's Disease* .. ... ... ... 1899

**Tweedie, Mrs. A.** [Ed.] *George Harley*... ... ... ... ... ... ... ... 1899

**Van Dieren, E.** ... *Beri-Beri en Voeding* ... ... ... ... ... ... ... 1898

**Van Valzah and J. D. Nisbet, W. W.** [Eds.] *The Diseases of the Stomach* 1899

**Vierordt, O.**... ... *Medical Diagnosis.* (Tr. by F. H. Stuart.) 4th Ed. 1898

**Walters, F. R.** ... *Sanatoria for Consumptives* ... ... ... ... ... ... 1899

**Williams, W. R.**... *Cancer* ... ... ... ... ... ... ... ... ... ... ... 1898

,, ... *Sarcoma* ... ... .. ... ... ... ... ... ... ... ... 1898

**Zuckerkandl, O.** ... *Atlas and Epitome of Operative Surgery.* (Translated. Ed. by J. C. DaCosta) ... ... ... ... ... 1898

---

### TRANSACTIONS, REPORTS, JOURNALS, &c.

American Journal of the Medical Sciences, The... ... ... Vol. CXVI. 1898
Annual and Analytical Cyclopædia of Practical Medicine ... Vol. II. 1899
Archives de Neurologie ... ... ... ... ... 2e Sér., Tomes V., VI. 1898
Birmingham Medical Review, The ... ... ... Vols. XLIII., XLIV. 1898
Brain ... ... ... ... ... ... ... ... ... ... ... ... ... Vol. X. 1888
British Almanac, The ... ... ... ... ... ... ... ... ... ... ... 1899
British Journal of Dental Science, The... ... (3) Vols. XVII.—XIX. 1874-76
British Journal of Dermatology, The ... ... ... ... ... ... Vol. X. 1898
British Medical Journal, The ... ... ... ... ... ... ... Vol. II. for 1898
Brooklyn Medical Journal, The .. ... ... ... ... ... ... Vol. XII. 1898
Cincinnati Lancet and Observer, The (3) Vols. IX.—XIII., 1866-70, XV. 1872
Directory of Booksellers, The International ... ... ... ... ... ... 1899
Dublin Journal of Medical Science, The ... ... ... ... Vol. CVI. 1898
Edinburgh Medical Journal, The ... ... ... ... ... N.S., Vol. IV. 1898
Edinburgh Obstetrical Society, The Transactions of the... Vol. XXII. 1897
Epidemiological Society of London, Transactions of the N.S., Vol. XVII. 1898
Glasgow Medical Journal, The ... .. ... ... ... ... ... Vol. L. 1898
Hazell's Annual ... ... ... ... ... ... ... ... ... 1886-91, 1893, 1899
Hospitals—

- Guy's Hospital Reports ... ... ... ... ... ... Vol. LIII. 1898
- Johns Hopkins Hospital Reports, The ... ... Vol. VII., No. 4. 1898
- Saint Bartholomew's Hospital Reports ... ... ...Vol. XXXIV. 1899
- Saint Thomas's Hospital Reports ... ... ...N.S., Vol. XXVI. 1898

Journal of Cutaneous and Genito-Urinary Diseases ... ... Vol. XVI. [1898]
Journal of the Sanitary Institute ... ... ... Vols. XV.—XVIII. 1894-97
Lancet, The... ... ... ... ... ... ... ... ... ... ... Vol. II. for 1898
Laryngological Society of London, Proceedings of the ... (2) Vol. V. 1898
Medical Directory, The ... ... ... ... ... ... ... ... ... ... ... 1899
Medical Press and Circular, The ... ... ... ... ... [Vol. CXVII.] 1898
Medical Record ... ... ... ... ... ... ... ... ... Vol. LIV. 1898
Medical Repository, The (New York) ... ... (3) Vols. III.—VI. 1800-1803
Medical Society of London, Transactions of the ... ... Vol. XXI. 1898
Medico-Chirurgical Transactions ... ... ... ... ... ...Vol. LXXXI. 1898

| | | |
|---|---|---|
| Mercy and Truth ... ... ... ... ... ... ... ... ... ... | Vol. II. | 1898 |
| Mexico. Informes Rendidos por los Inspectores Sanitarios de Cuartel y por los de los distritos al consejo Superior de Salubridad, [1896], 1897, 2 vols. ... ... ... ... ... ... ... ... ... ... | | 1898 |
| Michigan State Medical Society, Transactions of the ... | Vol. XXII. | 1898 |
| Odontological Society of Great Britain, Transactions of the | N.S., Vol. XXX. | 1898 |
| Philadelphia Medical Journal... ... ... ... ... ... ... ... ... | Vol. II. | 1898 |
| Practitioner, The ... ... ... ... ... ... ... ... ... ... | Vol. LXI. | 1898 |
| Retrospect of Medicine, Braithwaite's ... ... ... ... | Vol. CXVIII. | 1899 |
| St. Louis Medical and Surgical Journal, The | Vols. LXXIV., LXXV. | 1898 |
| Scottish Medical and Surgical Journal, The ... ... ... ... | Vol. III. | 1898 |
| West London Medical Journal ... ... ... ... ... ... ... | Vol. III. | 1898 |
| Whitaker's Almanack ... ... ... ... ... ... ... | 1876-82, 1884-92, | 1899 |
| Who's Who ... ... ... ... ... ... ... ... ... ... ... ... ... ... | | 1899 |
| Year-Book of Treatment, The... ... ... ... ... ... ... ... ... ... | | 1899 |

### Local Medical Notes.

### BRISTOL.

Hospital Sunday.—The second annual collection in places of worship on behalf of the Medical Charities of this City has been more productive than the first. This is partly due to better organisation, and partly to the fact that the recipients of the notes given for the collections found that they got more for their money by contributing through this Fund. The principle on which the distribution of notes was made was so arranged that the donors of small amounts received more in proportion than those who sent large ones, on the theory that the smaller subscriptions came from the poorer congregations. With very few exceptions the plan seems to have given satisfaction. It is hoped that next year, by arrangement with the Institutions, more notes will be at the disposal of the Committee. We cannot help expressing the great loss the Committee has experienced by the appointment of the Rev. Canon Cornish to the see of Grahamstown. It was largely due to his indefatigable exertions that the Hospital Sunday Fund was put upon its present sound basis, and the Committee will find it difficult to fill his place with a man of equal calibre, organising power, or enthusiasm.

The Convalescent Home.—This will soon, we hope, be opened for the reception of inmates. All the legal formalities have been concluded, and the necessary alterations are being pushed forward. No date has as yet been mentioned for the opening, but we believe that when the doors are opened the building will be found to be replete with every convenience.

The Prevention of Consumption.—A movement has been started to form a local committee or branch of the National Association for the Prevention of Consumption and other forms of Tuberculosis. Several meetings have been held to discuss the matter, and a provisional committee of medical men has been formed, of which Dr. E. L. Fox is the chairman; Dr. D. S. Davies, vice-chairman; Dr. Michell Clarke, honorary secretary; and Dr. P. Watson Williams, honorary treasurer. It is to be hoped that the citizens of Bristol will give their cordial assistance in this important matter, and that some practical results will follow.

University College.—Dr. G. Parker has been appointed Joint Lecturer on Medical Jurisprudence in place of Dr. A. J. Harrison, resigned.

Eye Dispensary.—Dr. Alex. Ogilvy has been appointed Surgeon to the Eye Dispensary in the place of Mr. Cyril H. Walker, resigned.

Fever Hospitals.—Dr. D. S. Davies has been appointed Medical Superintendent of the City of Bristol Fever Hospitals at Ham Green and Novers Hill.

